# Sophocarpine Attenuates LPS-Induced Liver Injury and Improves Survival of Mice through Suppressing Oxidative Stress, Inflammation, and Apoptosis

**DOI:** 10.1155/2018/5871431

**Published:** 2018-05-16

**Authors:** Zhengyu Jiang, Yan Meng, Lulong Bo, Changli Wang, Jinjun Bian, Xiaoming Deng

**Affiliations:** Faculty of Anesthesiology, Changhai Hospital, Second Military Medical University, 200433 Shanghai, China

## Abstract

Septic liver injury/failure that is mainly characterized by oxidative stress, inflammation, and apoptosis led to a great part of terminal liver pathology with limited effective intervention. Here, we used a lipopolysaccharide (LPS) stimulation model to simulate the septic liver injury and investigated the effect of sophocarpine on LPS-stimulated mice with endotoxemia. We found that sophocarpine increases the survival rate of mice and attenuates the LPS-induced liver injury, which is indicated by pathology and serum liver enzymes. Further research found that sophocarpine ameliorated hepatic oxidative stress indicators (H_2_O_2_, O_2_^∙^^−^, and NO) and enhanced the expression of antioxidant molecules such as superoxide dismutase (SOD), catalase (CAT), and glutathione (GSH). In addition, sophocarpine also attenuated regional and systematic inflammation and further reduced apoptosis of hepatocytes. Mechanistic evidence was also investigated in the present study as sophocarpine inhibited hepatic expression of the CYP2E/Nrf2 pathway during oxidative stress, inactivated p38/JNK cascade and NF-*κ*B pathway, and, meanwhile, suppressed PI3K/AKT signaling that reduced apoptosis. Conclusively, the present study unveiled the protective role of sophocarpine in LPS-stimulated oxidative reaction, inflammation, and apoptosis by suppressing the CYP2E/Nrf2/ROS as well as PI3K/AKT pathways, suggesting its promising role in attenuating inflammation and liver injury of septic endotoxemia.

## 1. Introduction

The liver plays a key role in immunological homeostasis and metabolism [1] while these crucial functions are usually impaired by lipopolysaccharide (LPS), inflammatory factors, and pathogens [2, 3]. LPS presents the major component of endotoxin in gram-negative bacteria and causes uncontrolled production of inflammatory mediators and oxidative stress, resulting in acute liver injury (and failure) [4]. LPS-induced liver injury in mice has been employed as a model for molecular pathological research [5], simulating the course of liver damage and failure in septic endotoxemia or sometimes septic shock or death [2, 6]. Liver failure is characterized by hepatic encephalopathy and disorder of protein synthesis [6]. Though specific mechanism remains controversial, consensus has been well reached that hepatic inflammatory oxidative stress and apoptosis might be the crucial mechanism.

Sepsis progression and septic liver dysfunction present complex pathophysiological alterations, [7], including processes like releasing of reactive oxygen species (ROS), nitrogen species (RNS), inflammation, and apoptosis. Characterized by the imbalance of endogenous enzymatic activity, such as catalase (CAT), superoxide dismutase (SOD), and glutathione (GSH) [8, 9], oxidative damage could be reflected by CYP2E1, which promotes the production of ROS during its catalytic cycle and may be the main contributor to oxidative stress and liver injury [10–12]. Thus, antioxidant compounds have been considered a promising treatment against ROS-induced liver injury or failure [13].

Additionally, oxidative stress is able to activate the p38 MAPK pathway, resulting in the activation of mitochondrial-related or other apoptotic pathways [14, 15]. In addition to the well acknowledged notion that phosphatidylinositol 3 kinase (PI3K)/Akt signaling as a key modulator of apoptotic process [16, 17], recent research proposed AKT could regulate apoptosis-related proteins including Bax, caspase-9, and caspase-3, which are also crucially involved in anticancer drug-stimulated apoptosis of cancer cells [18]. Nevertheless, the potential role of PI3K/AKT in liver injury remains unclear.

Liver injury induced by LPS is associated with inflammatory mediators including superoxide, nitric oxide, and tumor necrosis factor (TNF-*α*), interleukin-1*β* (IL-1*β*), interleukin-6 (IL-6), and other cytokines [19, 20]. LPS may activate the transcription factor nuclear factor-kappa B (NF-*κ*B) leading to activation of many inflammatory genes, such as TNF-*α* and IL-1*β* [19–21]. Therefore, inactivation of NF-*κ*B could attenuate LPS-induced sepsis and liver injury or liver failure.

Sophora alkaloids, such as sophocarpine, are able to be separated from the traditional Chinese herbs, for example, *Sophora flavescens* and *Sophora alopecuroides* [22, 23]. It has been reported that the extracts of *Sophora alopecuroides*, named Kudouzhi injection in clinical, have been used for the treatment of inflammation, pain, edema, and fever [23]. Furthermore, sophocarpine, called Kangke injection in clinical, has been employed to inhibit viral replication [24]. Sophocarpine also shows its activity against inflammation [23], and it suppresses inflammatory processes by inhibiting the JNK pathway, p38 MAPK pathway, and NF-*κ*B pathway, reducing the levels of iNOS and COX-2 [25]. However, the mechanisms underlying the role of sophocarpine remains unknown in LPS-induced liver injury.

In the present study, sophocarpine improved the liver function and protected the liver from inflammatory liver injury. To investigate its molecular mechanisms, the role of NF-*κ*B, PI3K/AKT, and CYP2E/Nrf2/ROS had been determined in acute liver injury. In the end, we evaluated the effects of sophocarpine on oxidative stress-associated apoptosis and inflammation in LPS-induced mice.

## 2. Materials and Methods

### 2.1. Cell Culture and Reagents

The hepatic stellate cells (HSCs) were purchased from ATCC (American Type Culture Collection, Manassas, VA, USA) and cultured in DMEM media supplemented with 100 U/mL penicillin and 100 mg/mL streptomycin (Gibco, Waltham, MA, USA) and 10% fetal bovine serum (FBS) (Gibco, Waltham, MA, USA) in a humidified incubator containing 5% CO_2_ and 95% air at 37°C. The HSCs were incubated with LPS (100 ng/mL) in the absence or presence of sophocarpine at the concentration of 1 *μ*M and 2 *μ*M about 24 h. These cells were used for the further studies.

### 2.2. Animals

Eight-week-old C57 BL/6 male mice, about 22.1 g per mouse, were employed for further study. And these mice were bought from the Experimental Animal Centre of Second Military Medical University (Shanghai, China) and were approved by the Animal Care and Use Committee of Changhai Hospital, Second Military Medical University (Shanghai, China). They were placed in a specific pathogen-free (SPF) room with sawdust bedding at a temperature of 25-26°C and a relative humidity of ~50% and light 12 h/day, and water and food were free to access. The authors confirmed that all animals received human care, and all animal experiments were conducted in accordance with the relevant guidelines and regulations. In this study, the normal mice were starved about 16 hours and they were divided randomly into four groups: con (control), LPS-induced group, and LPS-induced mice pretreated with sophocarpine (30 mg/kg body weight per day and 60 mg/kg body weight per day). For the establishment of liver injury in LPS-administrated mice with endotoxemia, the mice were injected intraperitoneally with LPS (5 mg/kg body weight) and we prepared 30 mice for each group above. Sophocarpine was administered orally once at 16:00~17:00 every day for 24 days. Meanwhile, we marked the number of dead mice for each group.

### 2.3. Analyses of Liver Function

Performed as the indicators of hepatic function, serum and liver levels of glutathione (GSH), alanine transaminase (ALT), alkaline phosphatase (ALP), and aspartate transaminase (AST) were analyzed by employing the biochemical kits from R&D Systems (Minneapolis, MN, USA).

### 2.4. Analyses of H_2_O_2_ and O_2_^∙^^−^ Production and ROS Levels in Liver of Mice

Hepatic levels of O_2_^∙^^−^ were measured using the chemiluminescence method [26]. Firstly, the weighed liver tissues of mice were homogenized in lysis buffer, pH 7.4, containing 10 mM EDTA as well as 20 mM HEPES. The samples were centrifuged for 10 min at 1000*g*, and, then, the aliquot of samples was incubated with a Krebs-HEPES buffer, pH 7.4, containing 5 mM lucigenin (Sigma, Shanghai, China) about 2 min at 37°C. Next, light emission data were obtained on a M200 PRO multifunctional microplate reader (TECAN, Switzerland), and the results were showed as mean light unit (MLU) min/mg protein. Levels of O_2_^∙^^−^ were measured by adding SOD (350 U/mL) to the medium according to the manufacturer's instruction (R&D Systems, Minneapolis, MN, USA). In addition, liver tissues were homogenized in normal saline, and the samples were treated with equal volume of cold methanol for 60 min in a 4°C icebox. Then, the samples were centrifuged for half an hour at 10000*g* and we obtained the supernatant for H_2_O_2_ evaluation using the biochemical kits from the R&D Systems (Minneapolis, MN, USA). Protein concentration was measured using the Bradford method, and BSA was employed as the standard.

### 2.5. Determination of IL-1*β*, TNF-*α*, and IL-16 by ELISA

The weighed liver tissues were put in a cold PBS buffer (pH 7.0) containing 0.002% sodium acid, 0.1 mg/mL soybean trypsin inhibitor, 2 mM PMSF, 10 nM EDTA, and 1.0 mg/mL BSA. The tissues were homogenated, and, then, the samples were incubated for 2 h in a 4°C refrigerator. For further assays, the supernatants were collected by centrifugation at 12000*g* for 10 min. IL-1*β*, TNF-*α*, and IL-16 levels in the supernatant of the serum and liver were measured using ELISA kits (Sigma, Shanghai, China).

### 2.6. Hematoxylin-Eosin Staining

The liver tissues from mice were fixed in 10% formalin, and the fixed specimens were processed to paraffin blocks, sectioned (5 *μ*m), and stained with hematoxylin-eosin (H&E) for histological analysis according to the standard protocols [27]. In this study, the sections were observed in a blind manner [27].

### 2.7. Reverse Transcription Polymerase Chain Reaction (RT-PCR)

The reverse transcription polymerase chain reaction (RT-PCR) and the quantitative real-time PCR (Q-PCR) were performed as previously described [20]. Total RNA was extracted from liver tissues and HSCs using TRIzol reagent from Thermo Fisher Scientific (Waltham, MA, USA). The cDNA was obtained by reverse transcription in a 20 *μ*L reaction containing 2 *μ*g of total RNA, oligo (dT), and reverse transcription premix.

The quantitative real-time PCR (Q-PCR) reactions were performed with the SYBR green PCR system in an ABI 7500 thermal cycler (Thermo Fisher Scientific, Waltham, MA, USA). The SYBR green reagents were also purchased from Thermo Fisher Scientific. The cycling conditions were as follows: 95°C for 3 min; followed by 40 cycles involving denaturing at 95°C for 10 s, annealing at 60°C for 5 s, and extension at 72°C for 10 s. Expression of mRNAs was normalized by the mRNA levels of *β*-actin, which was used as an internal control. The primers were shown here are PI3K, sense, 5′-TGACAGTAGGAGGAGGTTGG-3′, antisense, 5′-TCAGCCACATCAAGTATTGG-3′; AKT, sense, 5′-GAAGGTGATTCTGGTGAAAGAG-3′, antisense, 5′-ACACGGTTCTCAGTAAGCG-3′; caspase-9, sense, 5′-TCTTCATCTCCTGCTTAGAGG-3′, antisense, 5′-TGCTCCTTTGCTGTGAGTC-3′; caspase-3, sense, 5′-TGGAAAGCCGAAACTCTTC-3′, antisense, 5′-AGGAATAGTAACCAGGTGCTG-3′; caspase-6, sense, 5′-AGCGCGTACTTAAATGCAGAGG-3′, antisense, 5′-GTTGTAAGGTGGACAGGCTT-3′; Cyto-C, sense, 5′-CAGACAAGAAGAGGTTGCC-3′, antisense, 5′-CGTCATGGCAGTGTGTATTGG-3′; Bad, sense, 5′-CAGAGTTTGAGCCGAGTGAG-3′, antisense, 5′-TCCCTGCTGATGAATGTTG-3′; Bcl-xL, sense, 5′-GCAGGCGATGAGTTTGAAC-3′, antisense, 5′-TCCTTGTCTACGCTTTCCAC-3′; GAPDH, sense, 5′-CATTCAAGACCGGACAGAGG-3′, antisense, 5′-ACATACTGCACACCAGCATCACC-3′. In the end, we analyzed the relative levels of mRNAs using the 2^−ΔΔCt^ method and GAPDH was considered as the internal control.

### 2.8. Immunoblot Analysis

The liver tissues or the HSCs were lysed in RIPA Buffer (1 mM EDTA pH 8.0, 50 mM Tris-HCl pH 8.0, 2% SDS, and 5 mM DTT), and their protein concentration was decided by the BCA assay (Beyotime Inc., Shanghai, China). The total protein (about 30 *μ*g) was separated by a SDS-PAGE gel and transferred to PVDF (polyvinylidene fluoride) membranes (Invitrogen, CA, USA)= and blocked with 5% nonfat dry milk in PBST (phosphate-buffered saline with Tween), pH 7.5. The membranes were immunoblotted with primary antibodies for 4 hours or overnight at 4°C. The primary antibodies were all purchased from Cell Signaling Technology (MA, USA), and they were diluted at 1 : 1000 in the immunoblot analysis. Secondary antibodies with horseradish peroxidase were used in this study. The protein bands were determined by an enhanced chemiluminescence kit (Pierce, Rockford, USA). The corresponding semiquantitative analysis was based on optical density with ImageJ software.

### 2.9. Determination of the Apoptotic Cells by TUNEL

We determined the apoptosis of HSCs using TUNEL methods as previously described [19]. Briefly, the TUNEL and DAPI, which were from Sigma (Shanghai, China), were used to detect the apoptosis of cultured cells and the apoptotic cells could be TUNEL-positive. Then, the TUNEL-positive HSCs were calculated under a Carl Zeiss microscope (Axio Observer A1, Jena, Germany).

### 2.10. Statistical Analysis

Data were shown as the mean ± SEM. Student's *t*-test was performed for comparisons between two groups, and one-way ANOVA test was employed for comparisons among several groups. Log-rank test was used for survival data. *P* value < 0.05 was considered to be statistically significant.

## 3. Results

### 3.1. Sophocarpine Increases the Survival Rate and Attenuates the LPS-Induced Liver Injury

**Figure 1:**
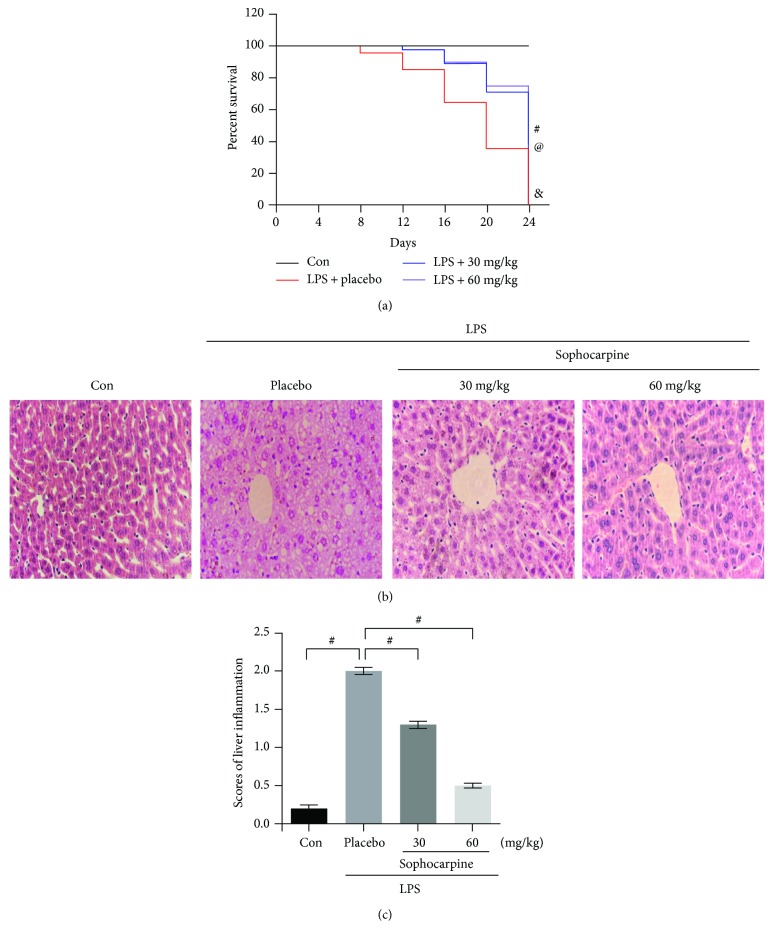
Sophocarpine ameliorates LPS-induced liver injury of mice. (a) Sophocarpine increased survival of endotoxemic mice induced by LPS; ^#^*P* < 0.001, the sophocarpine-treated group (30 mg/kg body weight per day) versus the LPS-induced group; ^@^*P* < 0.001, the sophocarpine-treated group (30 mg/kg body weight per day) versus the LPS-induced group; ^&^*P* < 0.001, the LPS-induced group (5 mg/kg body weight) versus the normal group. (b) Liver sections stained with H&E at a magnification of 200x. (c) Assessment of liver injury following portal inflammation scores; data are expressed as mean ± SEM, *n* = 30. ^#^*P* < 0.001.

The data in our study suggested that the 16-day survival rate was 73.3% (22 out of 30) and 76.7% (22 out of 30) in sophocarpine-pretreated group in a dose-dependent manner; meanwhile, the 16-day survival rate was 30.0% (9 out of 30) in the sepsis group (Figure 1(a)). Compared to the sepsis group, the 16-day survival rate was higher in the sophocarpine-treated group (*P* < 0.001); in the sham group (30 mice), the survival rate was 100% on the 16th day. In a word, pretreatment of mice with sophocarpine before LPS injection remarkably decreased lethality in contrast to LPS-caused sepsis animals.

**Figure 2:**
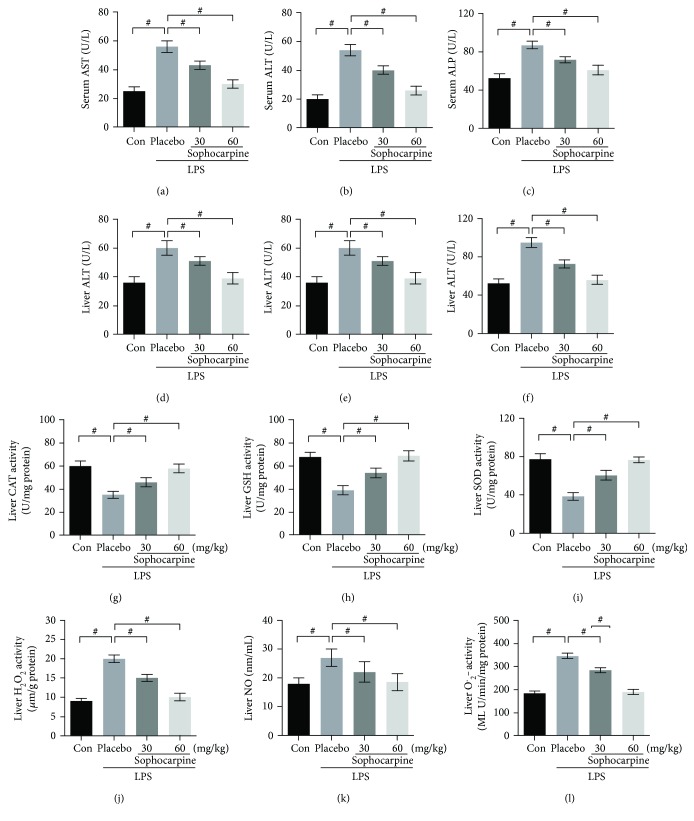
Sophocarpine reduces production of liver injury-related factors and improves oxidative stress in the liver of LPS-induced mice. (a–c) Sophocarpine decreased concentrations of AST, ALT, and ALP in serum determined by ELISA. (d–f) Sophocarpine downregulated levels of AST, ALT, and ALP in the liver analyzed by ELISA. The levels of CAT (g), GSH (h), SOD (i), H_2_O_2_ (j), NO (k), and O_2_^∙^^−^ (l) determined by ELISA. Data are expressed as mean ± SEM, *n* = 30. ^#^*P* < 0.001.

It has been reported that LPS-induced liver dysfunction may be assessed by serum liver-specific enzymes including AST, ALT, and ALP, and the morphological alterations of the liver may be observed by H&E staining. Firstly, we found that sophocarpine (30 mg/kg and 60 mg/kg per day) recovered destructive damage of hepatocytes significantly in LPS-induced septic liver injury (Figures 1(b) and 1(c)). Then, AST, ALT, and ALP levels in sepsis mice were higher than sham (normal) group, and sophocarpine significantly decreased AST, ALT, and ALP levels in the serum and liver of sepsis mice (Figures 2(a)–2(f)). Combined with the survival rate in Figure 1(a), the data revealed that sophocarpine showed its protective role in sepsis and sepsis-related acute liver injury via downregulating ALT, AST, and ALP expression.

### 3.2. Sophocarpine Ameliorates Oxidative Stress-Associated Indicators in LPS-Induced Mice

To demonstrate the effects of sophocarpine on oxidative stress in the liver of LPS-induced mice, we analyzed the antioxidative factors such as SOD, GSH, and CAT and detected the levels of H_2_O_2_, O_2_^∙^^−^, and NO in the liver. The results showed that activity of SOD, CAT, and GSH was decreased obviously in sepsis mice. As expected, sophocarpine (30 and 60 mg/kg) evidently restored CAT activity (Figure 2(g)), GSH activity (Figure 2(h)), and SOD activity (Figure 2(i)). Moreover, hepatic H_2_O_2_, O_2_^∙^^−^, and NO levels were determined in mice. The results indicated that LPS elevated the H_2_O_2_, O_2_^∙^^−^, and NO levels in the liver of mice, and sophocarpine significantly may suppress the H_2_O_2_, O_2_^∙^^−^, and NO production in the liver of LPS-administrated mice (Figures 2(j)–2(l)). Thus, sophocarpine prevented liver injury via attenuating ROS production and oxidative stress in LPS-induced mice.

### 3.3. The Effects of Sophocarpine on ROS Pathway in LPS-Induced Mice

**Figure 3:**
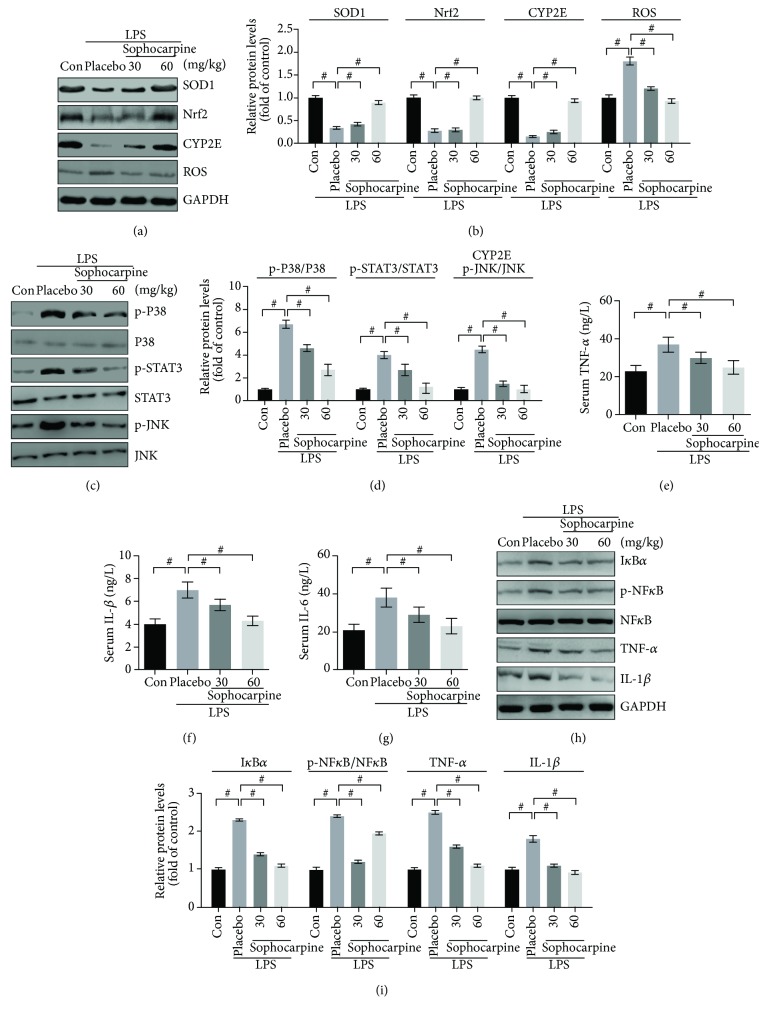
Effect of Sophocarpine on the expression of the hepatic oxidative stress-associated protein and inflammatory signaling pathway in the liver of LPS-induced mice. (a and b) Sophocarpine upregulated the expression of SOD1, Nrf2, and CYPE2 and downregulated the levels of ROS protein detected by Western blot (a) and the semiquantitative analysis of lanes was based on optical density with ImageJ software (b); data are expressed as mean ± SEM, ^#^*P* < 0.001. (c and d) Sophocarpine inhibited phosphorylation of P38, STAT3, and JNK and (c) and the corresponding semiquantitative analysis was based on optical density with ImageJ software (d). (e–g) Serum levels of TNF-*α*, IL-1*β*, and IL-6 analyzed by ELISA in mice; data are expressed as mean ± SEM, ^#^*P* < 0.001. (h and i) Sophocarpine blocked the expression of I*κ*B*α* and inactivated the NF-*κ*B; also, sophocarpine suppressed the expression of TNF-*α* and IL-1*β* demonstrated by Western blot (h). The semiquantitative analysis of lanes was based on optical density with ImageJ software (i); data are expressed as mean ± SEM, ^#^*P* < 0.001.

To investigate the inhibitory mechanism of sophocarpine against oxidative stress, we examined the ROS signaling in the LPS-induced liver of mice by Western blot. As shown in Figure 3, the results demonstrated that SOD1 and Nrf2 expression was dramatically downregulated in the LPS-induced liver, compared with the normal mice. After injection of sophocarpine, data presented that the levels of SOD1 and Nrf2 were elevated markedly by sophocarpine in a dose-dependent manner in endotoxic mice (Figures 3(a) and 3(b)). Moreover, we investigated oxidative stress-associated protein including ROS, CYP2E, P38, JNK, and STAT3 in mice. The data showed that ROS, CYP2E, P38, STAT3, and JNK were increased in LPS-induced mice (Figures 3(c) and 3(d)). However, the expression of CYP2E and ROS, as well as the phosphorylation of P38, STAT3, and JNK, was significantly inhibited by sophocarpine in a dose-dependent manner (Figures 3(c) and 3(d)). Thus, sophocarpine protected against endotoxemia via improving ROS-mediated oxidative stress in the liver of sepsis model animals.

### 3.4. Sophocarpine Attenuates Inflammation in LPS-Induced Liver Injury

As shown in Figures 3(e)–3(g), the LPS-induced mice exhibited higher content of serum IL-1*β*, TNF-*α*, and IL-6 compared with the normal mice. Compared with the LPS-induced group, sophocarpine administration (30 mg/kg and 60 mg/kg per day) significantly reduced serum levels of TNF-*α*, IL-1*β*, and IL-6 in LPS-induced mice. Similarly, we found that LPS enhanced the protein expression of TNF-*α* and IL-1*β*, and LPS also upregulated expression of I*κ*B*α* protein and phosphorylation of NF-*κ*B (Figures 3(h) and 3(i)). Contrarily, sophocarpine downregulated the expression of the above proteins in inflammation of the LPS-induced liver of mice (Figures 3(h) and 3(i)). The data indicated that sophocarpine may ameliorate LPS-induced liver injury by suppressing inflammation responses.

### 3.5. Sophocarpine Suppresses Apoptosis in Liver of LPS-Induced Mice

**Figure 4:**
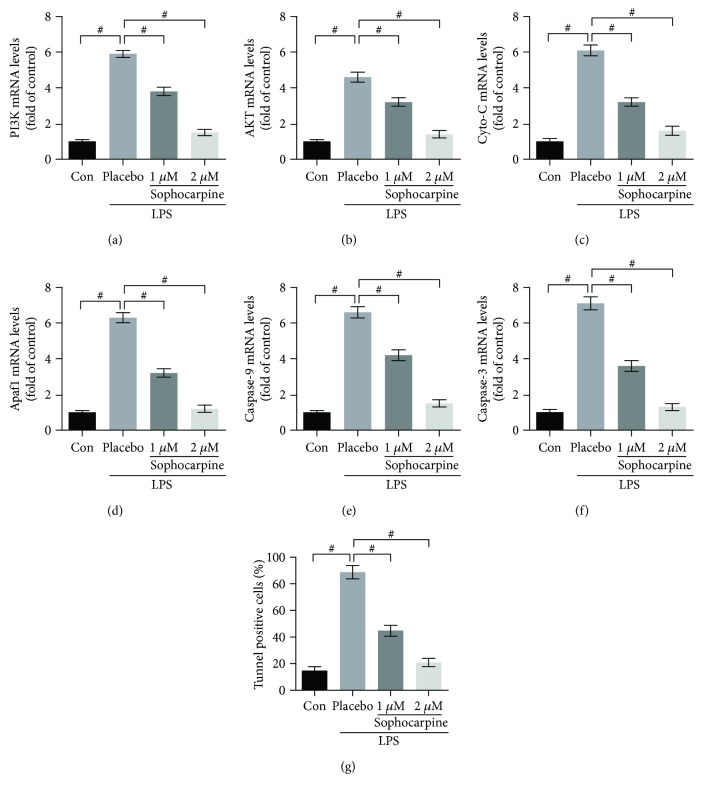
Sophocarpine improves injury of LPS-treated hepatic stellate cells (HSCs) by suppressing apoptosis. (a–f) Sophocarpine decreased the mRNA expression of PI3K, AKT, Cyto-C, Apaf1, caspase-9, and caspase-3 analyzed by real-time PCR; the data are expressed as mean ± SEM, ^#^*P* < 0.001. (g) Evaluation of apoptosis in HSCs by TUNEL assays and number of TUNEL-positive cells was calculated under a microscope; the data are expressed as mean ± SEM, ^#^*P* < 0.001.

**Figure 5:**
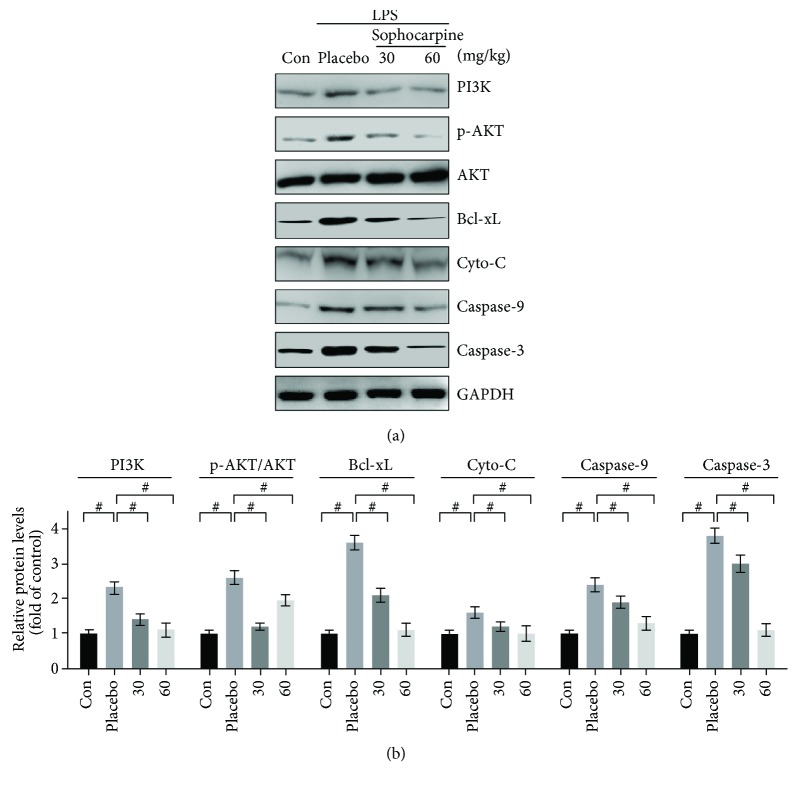
Sophocarpine represses apoptosis-associated signaling protein in the liver of LPS-induced mice. (a) Sophocarpine decreased the expression of PI3K, Bcl-xL, Cyto-C, caspase-9, and caspase-3 and depressed phosphorylation of AKT determined by Western blot. (b) The semiquantitative analysis of lanes was based on optical density with ImageJ software; data are expressed as mean ± SEM, ^#^*P* < 0.001.

To explore the effects of sophocarpine on apoptosis, we analyzed the PI3K/AKT pathway-related apoptosis progressing. The data proved that LPS significantly promoted the expression of PI3K and AKT, which were restored to the normal levels by administration of sophocarpine at 60 mg/kg per day. Furthermore, apoptosis-associated proteins were analyzed by Western blot in this section. Then, we found that Bcl-xL, Cyto-c, Apaf1, and cleaved caspase-9 and caspase-3 were increased by LPS dramatically (Figures 4(a) and 4(b)), indicating that LPS may promote apoptosis development in the liver of mice. After administrating sophocarpine, LPS-induced apoptosis in the liver may be significantly inhibited by depressing the expression of the related proteins above (Figures 5(a) and 5(b)).

**Figure 6:**
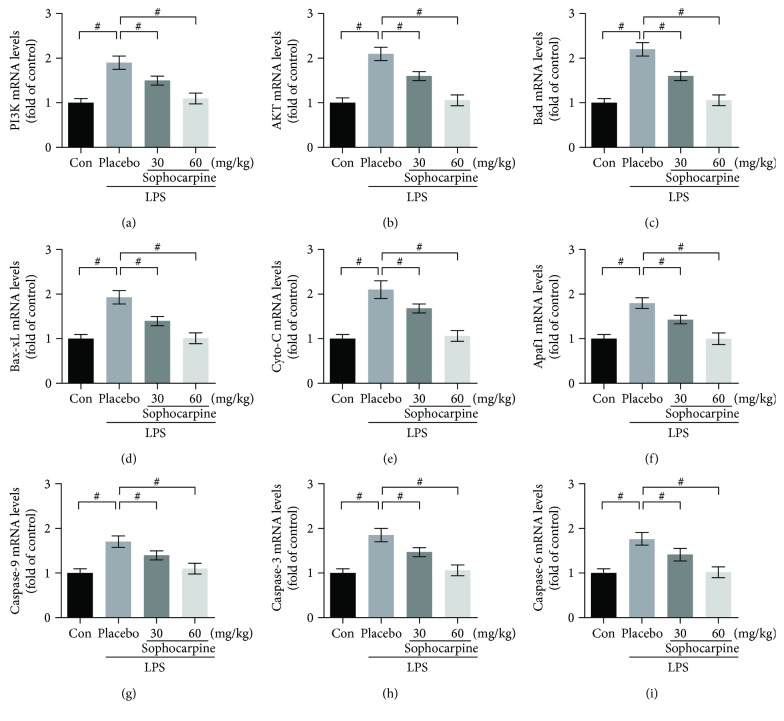
Sophocarpine suppresses apoptosis-associated gene mRNA levels in the liver of LPS-induced mice. Sophocarpine decreased the mRNA expression of PI3K (a), AKT (b), Bad (c), Bcl-xL (d), Cyto-C (e), Apaf1 (f), caspase-9 (g), caspase-3 (h), and caspase-6 (i) analyzed by real-time PCR. The data are expressed as mean ± SEM, ^#^*P* < 0.001.

To verify the vital role of apoptosis in the progression of LPS-induced acute liver injury, we analyzed the mRNA expression of the apoptosis-associated genes above by real-time PCR. The results showed that sophocarpine markedly downregulated the mRNA levels of PI3K and AKT (Figures 6(a) and 6(b)), and sophocarpine also reduced the mRNA levels of Bad, Bax-xL, Cyto-c, Apaf1, caspase-9, caspase-3, and caspase-6 in the liver of LPS-caused liver failure (Figures 6(c)–6(i)). Thus, sophocarpine attenuated liver injury by repressing the expression of apoptosis-related genes at both mRNA and protein levels.

### 3.6. Sophocarpine Improves Injury of LPS-Treated HSCs by Suppressing Apoptosis

To study the potential role of sophocarpine on LPS-stimulated hepatic stellate cells (HSCs), we pretreated HSCs with LPS. Then, the LPS-stimulated HSCs were subjected to sophocarpine incubation in order to determine whether sophocarpine may improve the liver injury by regulating apoptosis. In the present study, we found that LPS significantly elevated apoptosis-related gene expression, including PI3K and AKT, as well as Cyto-C, Apaf1, caspase-9, and caspase-3 (Figures 4(a)–4(f)). However, sophocarpine obviously inhibited this gene expression (Figures 4(a)–4(f)). Subsequently, sophocarpine may attenuate HSC damage and apoptosis in a dose-dependent manner (Figure 4(g)). These in vitro data suggested that sophocarpine could improve HSCs injury by suppressing PI3K/AKT-associated apoptosis.

## 4. Discussion

Nowadays, the mechanism of acute liver injury (or failure) still has not been completely investigated and required further study for promising clinical strategies [28]. Researches proposed LPS-induced acute liver injury, possibly derived from endotoxemia, was related to the inflammatory-associated Kupffer cells as well as inflammatory mediators including TNF-*α*, IL-1*β*, nitric oxide, and superoxide [29]. Furthermore, LPS extends acute liver injury by modulating the oxidative stress and production of free radical, protein synthesis, and apoptosis of hepatocytes [30]. In this study, we found that ALT, AST, and ALP were upregulated in the serum and liver of LPS-induced mice and these three indicators above were dramatically downregulated by sophocarpine (Figures 2(a)–2(f)).

Oxidative stress that is associated with cellular metabolism in the O_2_ environment has been regarded as a balance between prooxidant and antioxidant [31]. Based on the cellular microenvironment, the prooxidation process generates ROS including hydrogen peroxide (H_2_O_2_) and superoxide radical (O_2_^∙^^−^) [32]. The prooxidants H_2_O_2_, O_2_^∙^^−^, and NO are the main sources of ROS production according to the diverse stress conditions [33, 34]. Previous studies have demonstrated that the ROS plays a vital role in septic shock and organ failure [35]. Also, excessive ROS is expressed in LPS-induced liver injury and antioxidant agents seem to be a good choice to reverse the liver injury [35]. As a main antioxidant and O_2_^∙^^−^ scavenger, SOD may react with ROS and NO [36]. CAT may reduce ROS production by degrading H_2_O_2_ into oxygen and water [37]. Additionally, GSH can protect the liver and other organs against oxidative stress by decreasing the levels of H_2_O_2_ and lipid hydroperoxide [38]. In the current study, we found that LPS significantly elevated the levels of H_2_O_2_, O_2_^∙^^−^, and NO, which were downregulated nearly to normal levels by sophocarpine in a dose-dependent manner. Meanwhile, sophocarpine obviously upregulated the activity and expression of endogenous antioxidants, such as SOD, CAT, and GSH, suggesting that sophocarpine shows its potential antioxidative by blocking ROS-mediated signaling. As an essential sensor of redox status in ROS process, cellular Nrf2 often binds to the cytoskeletal-anchoring protein under normal conditions [39]. As the main contributor, CYP2E always promotes the production of ROS in liver injury and ethanol-induced oxidative stress [11]. In the present study, LPS decreased the levels of CYP2E, Nrf2, and ROS, which may be restored to normal by sophocarpine administration, indicating that sophocarpine possesses the activity to attenuate oxidative stress resulting in the improvement of LPS-triggered acute liver injury.

It has been reported that PI3K/Akt signaling plays vital roles in survival and antiapoptosis of cells by modulating its downstream targets, including caspase-9, caspase-3, and Bad [14, 34, 36]. Meanwhile, NF-*κ*B signaling has been demonstrated to be an important regulator in apoptosis of cancer cells [34, 36]. However, it remains unknown about the role of PI3K/Akt signaling in the liver of LPS-induced mice. In this study, sophocarpine significantly downregulated the expression of PI3K and phosphorylation of AKT, implying that the chemical can inhibit the expression of activated AKT. Furthermore, we found that sophocarpine reduced the expression of Bcl-xL, which was an antiapoptotic gene. Sophocarpine downregulated the expression of cleaved caspases substantially. Consequently, the data indicate that sophocarpine suppresses the activation of PI3K/AKT signaling resulting in apoptosis of liver injury induced by LPS.

NF-*κ*B signaling is a central regulator of inflammatory cytokines, such as TNF-*α* and IL-1*β*, which are key factors in inflammation responses [40]. In the current study, we found that LPS dramatically upregulated content and activity of TNF-*α*, IL-1*β*, and IL-6 in the serum and liver of mice. Thus, LPS-induced liver failure may be generated by promoting the inflammation responses including the mentioned inflammatory signaling and cytokines, which can be inhibited by sophocarpine in a dose-dependent manner.

In conclusion, sophocarpine shows the activity against oxidative stress and inflammation in the LPS-induced liver injury of mice. Moreover, sophocarpine suppresses the liver injury in LPS-induced mice with endotoxemia through blocking the inflammatory pathway NF-*κ*B, contributing to downregulation of proinflammatory cytokines. Meanwhile, sophocarpine affects apoptosis in the liver by inhibiting the PI3K/AKT-associated signaling. These findings suggest sophocarpine might be a novel and promising agent to improve inflammation, apoptosis, and oxidative responses in the liver of mice with endotoxemia.
