# Erratum to “Sophocarpine Attenuates LPS-Induced Liver Injury and Improves Survival of Mice through Suppressing Oxidative Stress, Inflammation, and Apoptosis”

**DOI:** 10.1155/mi/9842314

**Published:** 2025-09-10

Z. Jiang, Y. Meng, L. Bo, C. Wang, J. Bian, and X. Deng, “Sophocarpine Attenuates LPS-Induced Liver Injury and Improves Survival of Mice through Suppressing Oxidative Stress, Inflammation, and Apoptosis,” *Mediators of Inflammation* 2018 (2018): 5871431, https://doi.org/10.1155/2018/5871431.

In the article titled “Sophocarpine Attenuates LPS-Induced Liver Injury and Improves Survival of Mice Through Suppressing Oxidative Stress, Inflammation, and Apoptosis,” there was an error in Figure 3h. The image showing p-NFκB in Figure 3h is duplicated in the image of IκBα (Figure 3h). The corrected Figure 3h is shown below and is listed as Figure 1:

We apologize for this error.

## Figures and Tables

**Figure 1:**
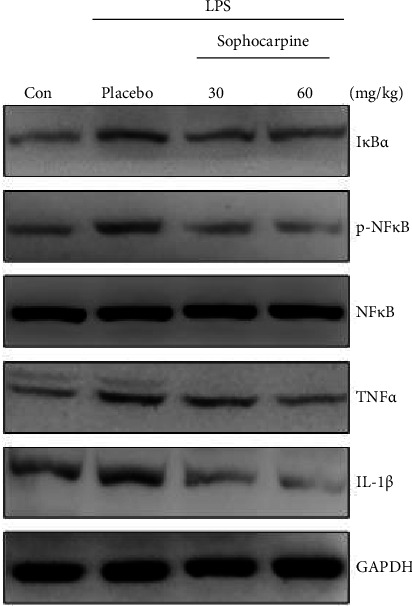
Effect of sophocarpine on the expression of the hepatic oxidative stress-associated protein and inflammatory signaling pathway in the liver of LPS-induced mice. (a, b) Sophocarpine upregulated the expression of SOD1, Nrf2, and CYPE2 and downregulated the levels of ROS protein detected by western blot (a) and the semiquantitative analysis of lanes was based on optical density with ImageJ software (b). Data are expressed as mean ± SEM, ^#^*p* < 0.001. (c, d) Sophocarpine inhibited phosphorylation of P38, STAT3, and JNK (c) and the corresponding semiquantitative analysis was based on optical density with ImageJ software (d). (e–g) Serum levels of TNF-α, IL-1β, and IL-6 analyzed by ELISA in mice. Data are expressed as mean ± SEM, ^#^*p* < 0.001. (h, i) Sophocarpine blocked the expression of IκBα and inactivated the NF-κB. Also, sophocarpine suppressed the expression of TNF-α and IL-1β demonstrated by western blot (h). The semiquantitative analysis of lanes was based on optical density with ImageJ software (i). Data are expressed as mean ± SEM, ^#^*p* < 0.001.

